# A Pharmacist's Contribution to Hospital Economy

**Published:** 1917-12-01

**Authors:** 


*To the Editor of* THE HOSPITAL.

### A Pharmacist's Contribution to Hospital Economy.

*To the Editor of* THE HOSPITAL.

SIR,—It may interest you to learn that, as a result of an article which appeared in THE HOSPITAL advocating economies in the pharmaceutical department, we have for the last six months at the hospital to which I am attached used a soft paraffin basis for making zinc ointment. During this period 2 cwt. of pure lard were saved for human consumption by this means, and incidentally the cost of manufacture was nearly halved.

The surgeons report favourably on the new preparation, and, in fact, for some cases prefer it.—Yours faithfully,

HOSPITAL PHARMACIST.

